# Adsorption of molybdenum by melanin

**DOI:** 10.1186/s12199-019-0791-y

**Published:** 2019-05-17

**Authors:** Wei Chen, Kazunori Hashimoto, Yasuhiro Omata, Nobutaka Ohgami, Akira Tazaki, Yuqi Deng, Lisa Kondo-Ida, Atsushi Intoh, Masashi Kato

**Affiliations:** 1 Department of Occupational and Environmental Health, Nagoya University Graduate School of Medicine, 65 Tsurumai-cho, Showa-ku, Nagoya, Aichi 466-8550 Japan; 2 Voluntary Body for International Health Care in Universities, Nagoya, Japan

**Keywords:** Melanin, Molybdenum (Mo), Molybdate, Langmuir

## Abstract

**Background:**

Melanin is detectable in various sense organs including the skin in animals. It has been reported that melanin adsorbs toxic elements such as mercury, cadmium, and lead. In this study, we investigated the adsorption of molybdenum, which is widely recognized as a toxic element, by melanin.

**Methods:**

Molybdenum level of the mouse skin was measured by inductively coupled plasma mass spectrometry. The pigmentation level of murine skin was digitalized as the L* value by using a reflectance spectrophotometer. An in vitro adsorption assay was performed to confirm the interaction between molybdenum and melanin.

**Results:**

Our analysis of hairless mice with different levels of skin pigmentation showed that the level of molybdenum increased with an increase in the level of skin pigmentation (L* value). Moreover, our analysis by Spearman’s correlation coefficient test showed a strong correlation (*r* = − 0.9441, *p* < 0.0001) between L* value and molybdenum level. Our cell-free experiment using the Langmuir isotherm provided evidence for the adsorption of molybdenum by melanin. The maximum adsorption capacity of 1 mg of synthetic melanin for molybdenum was 131 μg in theory.

**Conclusion:**

Our in vivo and in vitro results showed a new aspect of melanin as an adsorbent of molybdenum.

**Electronic supplementary material:**

The online version of this article (10.1186/s12199-019-0791-y) contains supplementary material, which is available to authorized users.

## Introduction

Melanin is a stable pigment that is widely found in various animals and plants [1]. Melanin is synthesized from melanocytes in various sense organs including the skin in animals [2]. Previous studies have shown the protective effects of melanin in the skin against ultraviolet light irradiation [3, 4]. Previous studies have also shown adsorption of toxic elements such as mercury, lead [5], cadmium [6], and barium [7] by melanin. However, there is a lack of evidence for interactions of melanin with various toxic elements strictly based on chemical theory.

We previously established constitutively activated RET, a receptor-type tyrosine kinase [8–10], hairless transgenic mice (HL-RET-mice) of lines 242 and 304, which have different levels of skin pigmentation [7]. We objectively evaluated the skin pigmentations levels for HL-RET-mice of lines 242 and 304 and wild-type hairless mice as L* values by using a reflectance spectrophotometer [7, 11]. Moreover, we newly showed the interaction of melanin with barium after showing that the level of barium increased with increases in the levels of pigmentation in the skin in the HL-RET-mice of lines 242 and 304 and wild-type hairless mice [7].

Molybdenum is widely recognized as a toxic element. In fact, previous studies showed that dysregulation of molybdenum was associated with neurological abnormalities [12], osteoporosis [13], and liver dysfunction [14]. At present, however, there is no chemical evidence directly showing an interaction between molybdenum and melanin.

In this study, we tried to clarify the association between melanin and molybdenum using the skin of hairless mice with different pigmentation levels. We then tried to strictly elucidate the interaction between molybdenum and melanin based on chemical adsorption theory.

## Materials and methods

### Mice

RET-transgenic hairless mice (HL-RET-mice) with constitutively different pigmentation were developed by crossing hairless mice (Hos:HRM) with RET-transgenic mice of lines 242 [3] and 304 [2], respectively, following the method previously described [11]. All of the mice were kept in the Animal Research Center of Nagoya University under the conditions of controlled temperature and humidity. Mice were used for digitalization of pigmentation levels in the skin and also for measurement of levels of molybdenum that had spontaneously accumulated in the skin.

### Digitalization for pigmentation levels of mouse skin

Previous studies showed that a reflectance spectrophotometer (CR-400; Konica Minolta Sensing Inc., Japan) can be used to evaluate the levels of skin pigmentation in mice as well as in humans [11, 15]. Following the methods, the pigmentation level of murine skin was digitalized as the L* value.

### Measurement of molybdenum level in mouse skin

Molybdenum level of the mouse skin was measured by inductively coupled plasma mass spectrometry (ICP-MS; 7500cx, Agilent Technologies) following the method previously described [16]. In brief, to measure the level of molybdenum that had spontaneously accumulated in the skin, dorsal skin was obtained and digested by the wet ashing method for ICP-MS.

### Batch adsorption assay using synthetic melanin

An adsorption assay using synthetic melanin (CAS # 8049-97-6, Nacalai Tesque Inc., Japan) was performed following the method described previously [17]. The stock solution of molybdenum was prepared by dissolving ammonium molybdate (Wako Pure Chemical Industries Ltd., Japan). To determine the contact time for equilibrium adsorption, 1 mg synthetic melanin was suspended in 400 μL molybdenum solution with the concentration of 115 μg molybdenum/mL. After incubation for 0, 5, 20, 60, 90, and 120 min, the suspension was centrifuged at 13,200 rpm for 10 min for solid-liquid separation. For adsorption kinetics and isotherm studies, 1 mg of synthetic melanin was incubated with 0.1, 1, 40, 150, 300, 600, 900, and 1200 μg of molybdenum/ml for 60 min followed by solid-liquid separation. The concentrations of molybdenum in the supernatant and the precipitate were measured by ICP-MS after ashing the samples.

### Langmuir model analysis of an equilibrium adsorption isotherm

The amount of molybdenum adsorbed by synthetic melanin was calculated by plotting Ce on the *x*-axis and Qe on the *y*-axis after confirming that the Langmuir adsorption isotherm model was suitable as shown previously [18]. To calculate the maximum adsorption capacity, Ce on the *x*-axis and Ce/Qe on the *y*-axis were plotted to draw Langmuir linear graph, and the predicting equation was obtained according the method previously described [19].

$$ \mathrm{QE}=V\left(\mathrm{Co}-\mathrm{Ce}\right)/\mathrm{M} $$

Qe: molybdenum adsorbed by melanin (μg/mg)

*V*: volume of molybdenum solution (0.4 mL)

Co: initial molybdenum concentration (μg/mL)

Ce: molybdenum concentration in the supernatant (μg/mL)

$$ \mathrm{Ce}/\mathrm{Qe}=\left(1/\mathrm{a}+\mathrm{Ce}\right)/\mathrm{Qmax} $$

*a*: adsorption equilibrium constant

Qmax: maximum adsorption capacity (μg/mg)

*M*: weight of melanin (1 mg)

### Statistical analysis

Multiple comparisons were performed using one-way analysis of variance (ANOVA) with Tukey’s test. The correlations were performed using Spearman coefficient. All statistical analyses were performed using SPSS 25.0 (IBM Corp., Armonk, NY, USA). *p* < 0.05 was considered statistically significant.

## Results

### Hairless mice with different skin pigmentation levels

**Fig. 1:**
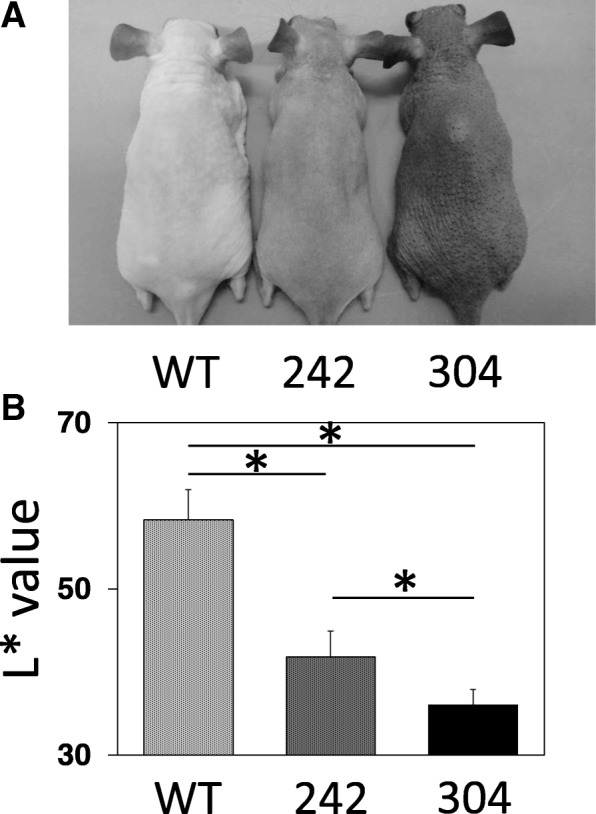
Hairless mice with different levels of skin pigmentation. **a** Photographs of dorsal skin from wild-type mice (WT) and HL-RET-mice of lines 242 (242) and 304 (304) at 4 months of age are shown. **b** The skin pigmentation levels (means ± SD, *n* = 11) in dorsal skin (L* values) are presented. Significant differences (**p < 0.01) were evaluated by the Tukey-Kramer test

Skin pigmentation levels of wild-type hairless mice and HL-RET-mice of lines 242 and 304 (Fig. 1a) were digitalized by using a reflectance spectrophotometer as the L* value according to the method previously shown [11]. As shown in Fig. 1b, the L* values in HL-RET-mice of lines 242 and 304 were decreased by 28% and 38%, respectively, compared to the L* value in wild-type mice, suggesting that the pigmentation levels in the skin of HL-RET-mice are increased.

### Correlation between levels of dorsal skin pigmentation and molybdenum

**Fig. 2:**
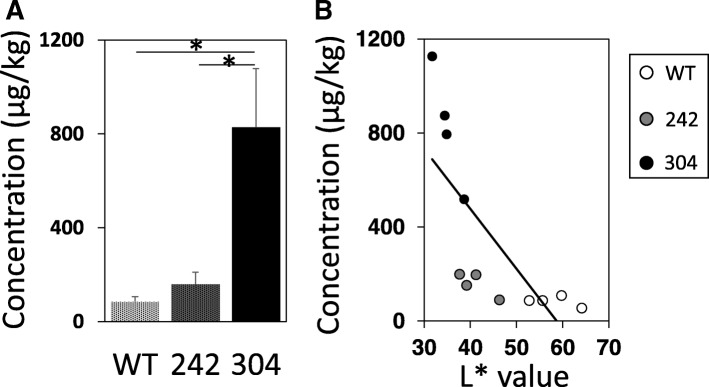
Correlation between levels of skin pigmentation and molybdenum in the skin. **a** Molybdenum levels (means ± SD) spontaneously accumulated in dorsal skin from wild-type hairless mice (WT, *n* = 4) and HL-RET-mice of lines 242 (242, *n* = 4) and 304 (304, *n* = 4) at 4 months of age are presented. Significant differences (**p < 0.01) were evaluated by the Tukey-Kramer test. **b** Correlations between levels of skin pigmentation (L* values) and levels of molybdenum spontaneously accumulated in dorsal skin of wild-type hairless mice (WT, *n* = 4) and HL-RET-mice of lines 242 (242, *n* = 4) and 304 (304, *n* = 4) at 4 months of age are shown. Results analyzed by Spearman’s rank correlation coefficient are presented due to no normal distribution

The levels of molybdenum that had spontaneously accumulated in the dorsal skin of HL-RET-mice of lines 242 and 304 were 1.9-fold and 9.8-fold higher, respectively, than the level in wild-type mice (Fig. 2a). Moreover, there was a significant correlation (*r* = − 0.9441, *p* < 0.0001) between the L* values of wild-type hairless mice and HL-RET-mice of lines 242 and 304, and the levels of molybdenum that had spontaneously accumulated in the dorsal skin by Spearman’s correlation coefficient test (Fig. 2b).

### Adsorption of molybdenum by synthetic melanin

**Fig. 3:**
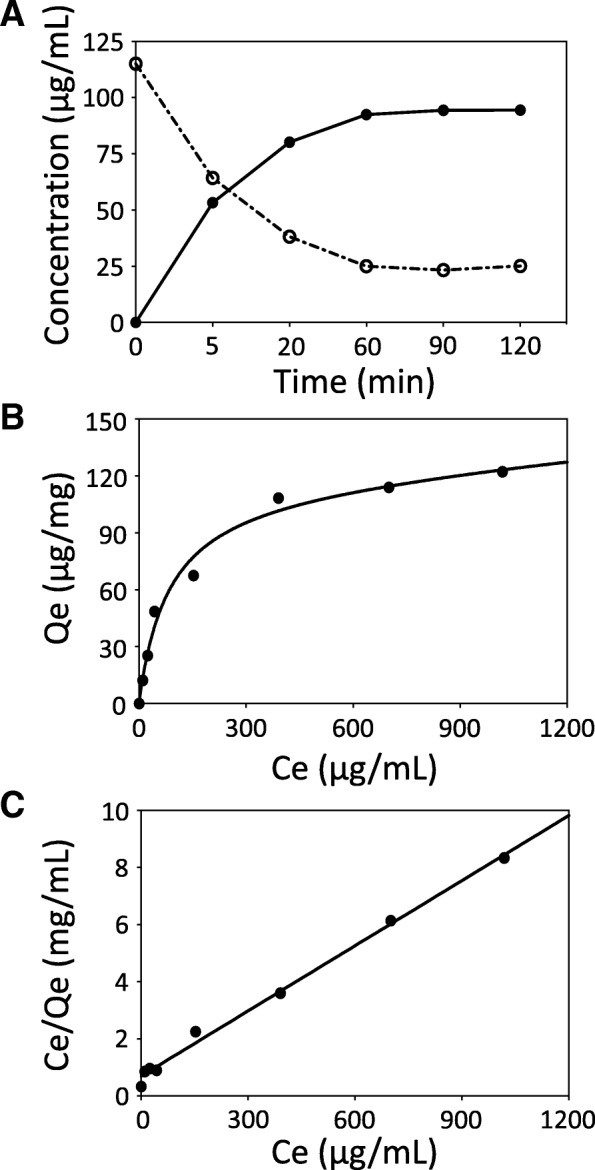
Adsorption of molybdenum by synthetic melanin. Levels of melanin-bound molybdenum (closed circle) and unbound molybdenum (open circle) molybdenum incubated in water for the indicated time (**a**) and equilibrium data (**b**) and Langmuir isotherm (**c**) for adsorption of molybdenum by synthetic melanin are presented. Consistent results were obtained by the experiment independently repeated three times, and representative result results are presented. Qe (μg/mg), amount of molybdenum adsorbed by melanin at equilibrium; Ce (μg/ml), concentration of molybdenum in solution at equilibrium; Qmax (131 μg/mg), maximum adsorption capacity

Interaction between molybdenum and melanin was then examined on the basis of the chemical adsorption theory. Concentrations of melanin-bound molybdenum and unbound molybdenum are shown with time courses in Fig. 3a. The equilibrium adsorption isotherm was obtained after 60-min incubation and was analyzed by the Langmuir adsorption isotherm model (Fig. 3b, c). The maximum adsorption capacity of synthetic melanin for molybdenum was 131 μg/mg in theory (Fig. 3c).

## Discussion

We demonstrated that molybdenum levels spontaneously accumulated in the strains of mice with different levels of skin pigmentation were correlated with levels of digitalized skin pigmentation (L* values). Our previous study showed a correlation (*r* = − 0.54) between barium levels and levels of digitalized skin pigmentation expressed as L* values after preparing genetically modified hairless mice with different pigmentation levels [7]. We then proved an interaction between synthetic melanin and barium in vitro according to the chemical adsorption theory using the Langmuir isotherm [7]. Since Spearman’s correlation coefficient (*r* = − 0.9441) between molybdenum levels and L* values was stronger than that between barium concentrations and L* values, direct evidence of an interaction between synthetic melanin and molybdenum in vitro was again obtained in this study. Our results suggest that correlations between L* values and elements might be a useful screening system for detecting elements adsorbed by melanin as a primary screening.

Previous studies showed that the maximum adsorption capacities (Qmax) of mercury and lead to synthetic melanin synthesized from *Pseudomonas stutzeri* were 82.4 μg/mg and 147.5 μg/mg, respectively [5]. A previous study showed that melanin purified from squid ink maximumly adsorbed 19.6 μg of chromium/mg [20]. Indole-5, 6-quinone unit-based synthetic melanin maximumly adsorbed 38.5 μg of barium/mg [7]. Our results showing 131 μg of molybdenum/mg as the maximum adsorption capacity of synthetic melanin may be reasonable compared with the other adsorbents previously reported as shown in Additional file 1. Thus, synthetic melanin is a potential candidate for an adsorbent of molybdenum. It remains unclear whether adsorption of molybdenum by melanin biologically plays a beneficial role or not. Further study is needed to clarify the biological significance of adsorption of molybdenum by melanin.

## Conclusion

This study demonstrated a strong correlation between levels of skin pigmentation and molybdenum in murine skin. Our cell-free analysis then showed adsorbed molybdenum by melanin using the Langmuir isotherm. Thus, this study chemically showed a new aspect of melanin as an adsorbent of molybdenum.

## Additional file

Additional file 1: Maximum adsorption capacities of melanin species for metal elements. (PPTX 39 kb)

## Abbreviations

HL: Hairless
ICP-MS: Inductively coupled plasma mass spectrometry
