# Supplementary material for: Adsorption of molybdenum by melanin
Source: Environ Health Prev Med. 2019 May 17;24:36. doi: 10.1186/s12199-019-0791-y (PMC6525471; doi:10.1186/s12199-019-0791-y)
Supplement: Supplementary file 1 — Maximum adsorption capacities of melanin species for metal elements. (PPTX 39 kb) [file 12199_2019_791_MOESM1_ESM.pptx]

## Slide 1
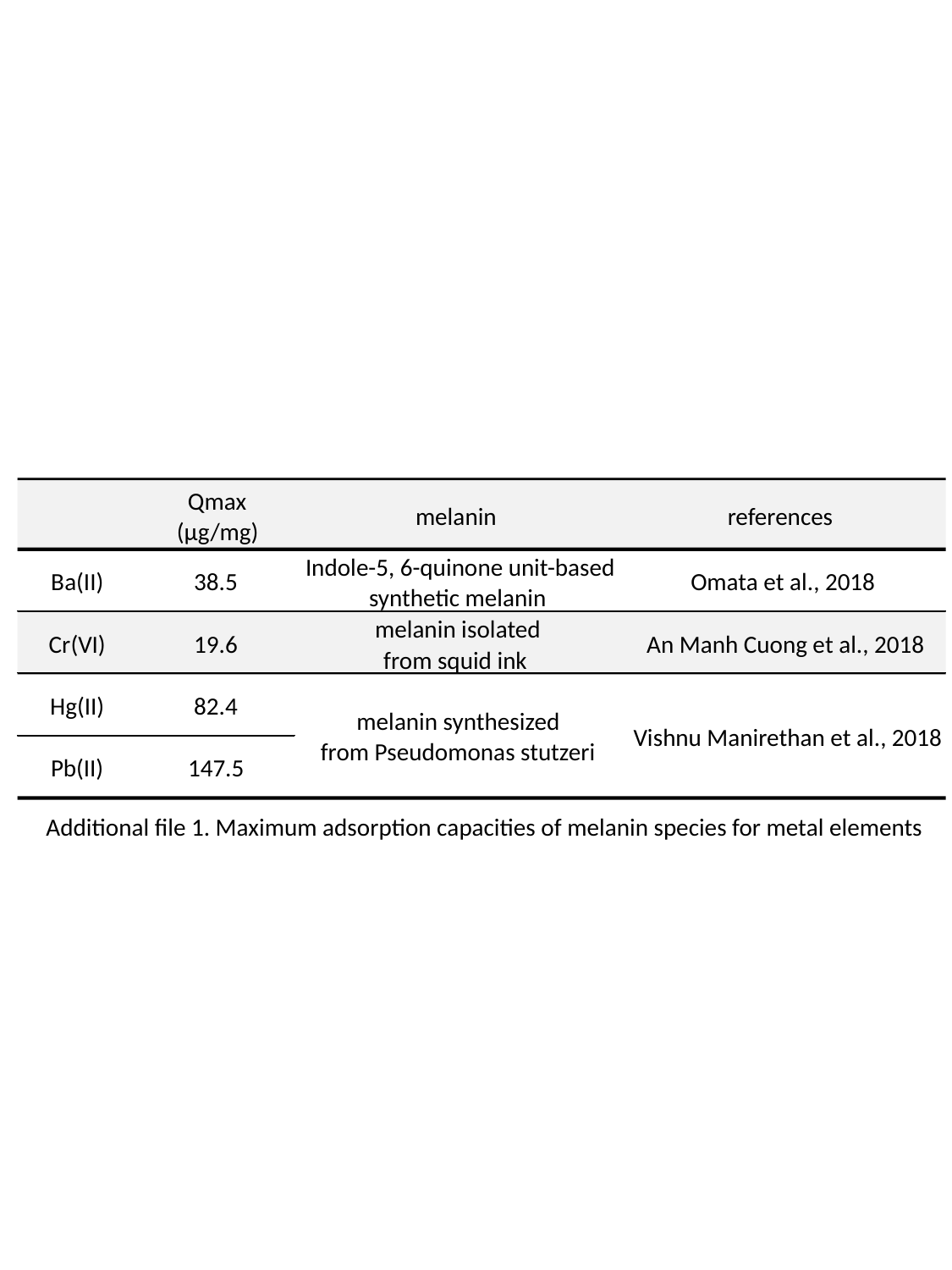

Qmax
melanin
references
(μg/mg)
Indole-5, 6-quinone unit-based
Ba(II)
38.5
Omata et al., 2018
synthetic melanin
melanin isolated
Cr(VI)
19.6
An Manh Cuong et al., 2018
from squid ink
Hg(II)
82.4
melanin synthesized
Vishnu Manirethan et al., 2018
from Pseudomonas stutzeri
Pb(II)
147.5
Additional file 1. Maximum adsorption capacities of melanin species for metal elements
